# Training needs and supports for evidence-based decision making among the public health workforce in the United States

**DOI:** 10.1186/s12913-014-0564-7

**Published:** 2014-11-14

**Authors:** Rebekah R Jacob, Elizabeth A Baker, Peg Allen, Elizabeth A Dodson, Kathleen Duggan, Robert Fields, Sonia Sequeira, Ross C Brownson

**Affiliations:** Prevention Research Center in St. Louis, Brown School, Washington University in St. Louis, St. Louis, MO USA; College for Public Health & Social Justice, Saint Louis University, St. Louis, MO USA; Division of Public Health Sciences and Alvin J Siteman Cancer Center, Washington University in St. Louis School of Medicine, St. Louis, MO USA

**Keywords:** Evidence-based decision making, Public health, Evidence-based practice, Public health workforce

## Abstract

**Background:**

Preparing the public health workforce to practice evidence-based decision making (EBDM) is necessary to effectively impact health outcomes. Few studies report on training needs in EBDM at the national level in the United States. We report competency gaps to practice EBDM based on four U.S. national surveys we conducted with the state and local public health workforce between 2008 and 2013.

**Methods:**

We compared self-reported data from four U.S. national online surveys on EBDM conducted between 2008 and 2013. Participants rated the importance of each EBDM competency then rated how available the competency is to them when needed on a Likert scale. We calculated a gap score by subtracting availability scores from importance scores. We compared mean gaps across surveys and utilized independent samples t tests and Cohen’s d values to compare state level gaps. In addition, participants in the 2013 state health department survey selected and ranked three items that “would most encourage you to utilize EBDM in your work” and items that “would be most useful to you in applying EBDM in your work”. We calculated the percentage of participants who ranked each item among their top three.

**Results:**

The largest competency gaps were consistent across all four surveys: economic evaluation, communicating research to policymakers, evaluation designs, and adapting interventions. Participants from the 2013 state level survey reported significantly larger mean importance and availability scores (p <0.001, d =1.00, and p <0.001, d = .78 respectively) and smaller mean gaps (p <0.01, d = .19) compared to the 2008 survey. Participants most often selected “leaders prioritizing EBDM” (67.9%) among top ways to encourage EBDM use. “EBDM training for specific areas” was most commonly ranked as important in applying EBDM (64.3%).

**Conclusion:**

Perceived importance and availability of EBDM competencies may be increasing as supports for EBDM continue to grow through trends in funding, training, and resources. However, more capacity building is needed overall, with specific attention to the largest competency gaps. More work with public health departments to both situate trainings to boost competency in these areas and continued improvements for organizational practices (leadership prioritization) are possible next steps to sustain EBDM efforts.

## Background

Applying an evidence-based framework to public health practice is essential for effective program and policy planning, implementation, and evaluation and has the potential for improved population health outcomes [1,2]. An evidence-based public health approach requires practitioners to utilize the best available research evidence, use data and information sources systematically, apply appropriate planning frameworks for programs or policies, engage community members in assessment and decision making processes, conduct sound evaluation of programs, and disseminate findings to key stakeholders [3–5]. Using this framework to guide policy or program decisions in public health is referred to as evidence-based decision making (EBDM) [2,4].

In order to engage in EBDM one needs a specified mix of knowledge, understanding and skills, or “competencies” [6] for EBDM processes. Main competencies needed for EBDM include action planning, adapting interventions, communicating research to policy makers, economic evaluation, designing evaluations (including qualitative and quantitative approaches), and prioritizing program and policy options [4]. Several of these key competencies are reflected in the Council on Linkages (COL) as being important for public health workforce development and education [7]. For example, in Financial Planning and Management Skills (COL’s domain 7), understanding and using cost-benefit and cost-utility analysis for program decision making mirrors the EBDM competency to incorporate economic evaluation into public health program decision making [7,8]. Similarly, EBDM competencies are consistent with the Public Health Accreditation Board (PHAB) standards for local and state public health agencies [9]. For example, public health agencies are charged to “identify and use the best available evidence for making informed public health practice decisions” (standard 10.1) and to “promote understanding and use of research results, evaluations, and evidence-based practices with appropriate audiences” (standard 10.2) [9]. While there have been studies to examine the use of some of these competencies in general [10,11], few studies have specifically examined the perceived competencies needed to engage in EBDM among the public health workforce.

The recognition of the importance of these competencies has led to the development of a variety of tools, resources and trainings to support the application of EBDM. For example, publicly available online tools such as the Community Guide [12], Cancer P.L.A.N.E.T. [13], Research to Reality [14], and the Community Toolbox [15] provide toolkits and resources to enhance these competencies. In addition, national agencies provide technical assistance (such as that offered through the National Association of Chronic Disease Directors (NACDD) State Technical Assistance & Review program) and recommendations for local and state agencies to more effectively implement EBDM [16]. Also, in-person trainings are offered to public health professionals to build capacity to practice core EBDM competencies through a series of stepped modules [17–19]. For example, a module in economic evaluation prepares attendees to incorporate cost-benefit and cost-utility analysis into decision making [19].These evidence-based resources and supports reflect the increasing awareness and perceived value of EBDM in academia, public health practice and funding organizations. However, little is known regarding the impact these have had on the perceived competency to engage in EBDM.

Identifying training needs for EBDM competencies is crucial to understand where more competency-based efforts to build capacity could be targeted through training, use of analytic tools, and other resources. However, representative, national level data on EBDM competencies are lacking. To address this gap, we compare perceptions of EBDM competencies reported in four U.S. national surveys we conducted with the public health workforce at the local and state level.

## Methods

We conducted four U.S. national online surveys of the state and local public health workforce. The main objective of the current study is to compare and contrast perceptions of EBDM competencies across these surveys. Participants were state and local public health department employees. Participants in all four surveys answered questions on ten competencies needed for EBDM. The four national sets of participants included in the current study are as follows. The first group is 441 U.S. state health department chronic disease prevention staff that completed a 2008 survey that assessed barriers to practice EBDM at the state level. The second group of respondents is 904 U.S. state health department chronic disease prevention staff from a 2013 survey that examined perceptions of state level support to practice EBDM. The third group is 517 local health department directors that completed a 2012 survey and the fourth group of respondents included in the current study is from a 2013 survey with 332 local health department program managers identified by the third group. The third and fourth groups of participants received the same survey, which sought to examine organizational support and barriers for EBDM at the local level. We describe the survey methods in the paragraphs that follow.

### State health department 2008 survey

In 2008, we administered an online survey with state level staff working in chronic disease prevention in all 50 states, District of Columbia, and U.S. territories. The 74-item survey derived from previous work [20,21] and included questions about use of the Community Guide and other resources, the application of evidence-based interventions, personal chronic disease-related health behaviors of the participants, and other demographic information. The survey underwent cognitive response testing (n =12) with experts in chronic disease prevention [22,23], and their feedback was incorporated into the final survey. The survey took participants approximately 15 minutes to complete.

All NACDD members were contacted via email to explain the survey’s purpose. A week later, participants received another email with a link to the online survey. Research staff completed follow-up via telephone and email until the survey closed in August 2008. As an incentive, participants were entered into a drawing to win one of numerous $20 gift cards or 25,000 airline miles. Of the 469 total participants (65% response rate), we included in this study the 441 state health department staff working in the 50 U.S. states and District of Columbia. The Saint Louis University Institutional Review Board reviewed and granted human subjects approval. Further details on the sampling methods and data collection are reported elsewhere [24].

### State health department 2013 survey

We administered an online survey in spring of 2013 with staff working in chronic disease prevention at the state level in all U.S. states and U.S. territories. The 68-item survey tool was based on our previous work in the 2008 survey mentioned above [5,24–26] and included participant characteristics, use of evidence-based interventions, presence of organizational supports for EBDM, and access to evidence resource. The survey was revised based on cognitive response testing interviews with chronic disease prevention experts that previously worked in state health departments (n =11) and reliability test-retest with 75 current state health department employees in chronic disease prevention. The survey took the participants approximately 15 minutes to complete.

Research staff identified a list of program managers and staff chronic disease prevention through state health department websites, NACDD contact lists, and contact lists from partnering organizations. Contacts from the list were sent email invitations with a survey link in Qualtrics [27]. Participants were offered an optional $20 gift card to Amazon.com upon completion. Of the 923 total participants (76% response rate), we included in this study the 904 state health department staff working in the 50 U.S. states and District of Columbia. The Institutional Review Board of Washington University in St. Louis reviewed and granted human subjects approval. Further details on the sampling methods and data collection are reported elsewhere [28].

### Local public health 2012–2013 surveys

Between December of 2012 and February of 2013, we conducted two surveys with local health departments; the first with local health department directors and the second with program managers. For both groups, we used a 66-item survey tool similar to that used in the 2013 state level survey and was based on previous work [8,17,21,24,29]. In addition, the tool included items based on a public health systems logic model and related frameworks [30–33] and assessed views on barriers to and management practices for EBDM among other items.

We selected a random sample (stratified by jurisdictional population size) of 1,067 local health departments from the National Association of County and City Health Officials database. Project staff sent survey links in Qualtrics [27] to the director of each randomly selected health department. There were 517 (54%) directors who completed the survey and are included in this study. The survey asked the participating directors to identify program managers within their local health department in chronic disease, environmental health, and infectious disease. We then sent the same online survey to each identified program manager directly, of which 332 completed the survey (67% response rate) and are included in this study. Participants were offered a $20 gift card to Amazon.com upon completion. The Institutional Review Board of Washington University in St. Louis reviewed and granted human subjects approval. Further details on the sampling methods and data collection are reported elsewhere [34–36].

### EBDM competency and supports measures

Each of the surveys described above asked participants to rate perceived importance and availability of ten specific EBDM competency items. Participants first rated the “importance of each of the skills to you” then rated “how available each skill is to you when you need it either in your own skill set or among others’ in your agency”. Both were rated on an 11-point Likert scale 0 = not important to 10 = very important and 0 = not available to 10 = very available. A total of eight EBDM competency items were identical or highly similar (same basic concept with only minor wording differences) across the surveys. These eight EBDM competency items are included in this study: action planning, adapting interventions, communicating research to policy makers, economic evaluation, evaluation designs, prioritization of program and policy options, qualitative evaluation, and quantitative evaluation.

In the 2013 state health department survey, participants also ranked three incentives that “would most encourage you to utilize EBDM in your work” and three training modalities that “would be most useful to you in applying EBDM in your work” from provided response option lists. Participants could also type in and rank “other” responses.

### Analysis

To summarize participant characteristics, we conducted descriptive analyses of participants in each of the four survey groups. Following previously utilized methods [29], we created a competency gap score by subtracting the Likert scale rated availability of each EBDM competency from rated importance. For example, a score of 9 for importance minus a score of 6 for availability equaled a gap score of 3. We calculated confidence intervals at 95% for the mean scores of rated importance, availability, and corresponding gaps. To compare state level participants’ competency gaps, we conducted independent samples t tests. Large sample sizes are prone to produce statistically significant results for small differences that may or may not have practical significance [37]. To address this, we calculated Cohen’s d values for mean differences observed in t tests. Cohen’s d values are calculated as the mean difference between samples divided by the pooled standard deviation from both samples [38]. Cohen’s d values give additional detail regarding the magnitude and direction of observed differences. Cohen suggests the following effect ranges for d: 0.2 small, 0.5 medium, and 0.8 large [38,39]. We aggregated mean gaps by survey, and created bar graphs to assess broader trends across all surveys. For each EBDM support rank item, we calculated the percentage of participants who ranked that item among their top three items. We restricted analysis to participants practicing in the 50 U.S. states and the District of Columbia which excluded 28 participants in the 2008 state level survey and 19 from the 2013 state level survey.

## Results

### Participant characteristics

Table 1 shows participant characteristics across the four respondent groups. There were higher percentages of females represented in the two state level surveys (approximately 80%) as compared to the local public health workforce surveys (between 60-65%). On average, participants in the local public health workforce surveys reported more years in their current position (7.7 for local program managers and 8.5 years for local directors) than those from the state level surveys (4.6 for 2008 and 4.9 for 2013 state level participants). Participants from the local directors survey had the highest mean years involved in public health (20.4 years). Local public health program managers had the lowest proportion of workforce reporting a Master in Public Health degree (18.1%).

**Table 1:** **Participant characteristics from four national surveys of state and local public health department professionals**

	**State**		**Local**	
	**2008 State health department staff (n =441)**	**2013 State health department staff (n =904)**	**2012 Local public health directors (n =517)**	**2013 Local public health program managers (n =332)**
	**n (%)**	**n (%)**	**n (%)**	**n (%)**
Gender				
Female	351 (80.0)	727 (80.4)	315 (60.9)	216 (65.1)
Male	88 (20.0)	177 (19.6)	202 (39.1)	111 (33.4)
Missing	2 (0.0)	0 (0.0)	0 (0.0)	5 (1.5)
Age				
20-29	19 (4.3)	64 (7.1)	8 (1.5)	17 (5.1)
30-39	76 (17.3)	214 (23.8)	44 (8.5)	60 (18.1)
40-49	118 (26.8)	248 (27.6)	110 (21.3)	77 (23.2)
50-59	177 (40.2)	246 (27.4)	228 (44.1)	121 (36.4)
60+	50 (11.4)	127 (14.1)	127 (24.6)	52 (15.7)
Missing	1 (0.0)	5 (0.0)	0 (0.0)	5 (1.5)
Years at agency Mean (SD)	10.5 (7.9)	9.9 (7.9)	---^a^	---^a^
Years in position Mean (SD)	4.6 (4.1)	4.9 (4.9)	8.5 (7.3)	7.7 (6.8)
Years in public health Mean (SD)	15.3 (8.6)	14.7 (9.2)	20.4 (14.6)	15.4 (9.2)
MPH/MPHS degree	114 (25.9)	275 (30.4)	136 (26.3)	60 (18.1)
Master’s degree or above^b^	332 (75.3)	632 (69.9)	186 (63.8)	142 (42.8)
Position				
Generalist^c^	344 (78.0)	577 (63.8)	500 (96.7)	265 (79.8)
Specialist^d^	54 (12.2)	264 (29.2)	3 (0.1)	34 (10.2)
Other^e^	17 (3.9)	63 (7.0)	14 (2.7)	28 (8.4)
Missing	26 (5.9)	0 (0.0)	0 (0.0)	5 (1.5)

*Abbreviations: SD* standard deviation, *MPH* Master of Public Health, *MPHS* Master of Public Health Sciences.

^a^Question was not asked in the surveys with local public health practitioners.

^b^Any master’s degree, including MPH, was included in this grouping.

^c^Generalists were grouped by program managers/administrators/coordinators, program planners, division or bureau heads, division deputy directors, department heads, or academic educators.

^d^Specialists were grouped by health educators, epidemiologists, statisticians, program evaluators, community health nurses, social workers, dietitians, or nutritionists.

^e^Other primary position (e.g. policy analyst, health inspector).

### Gap analyses

**Figure 1:**
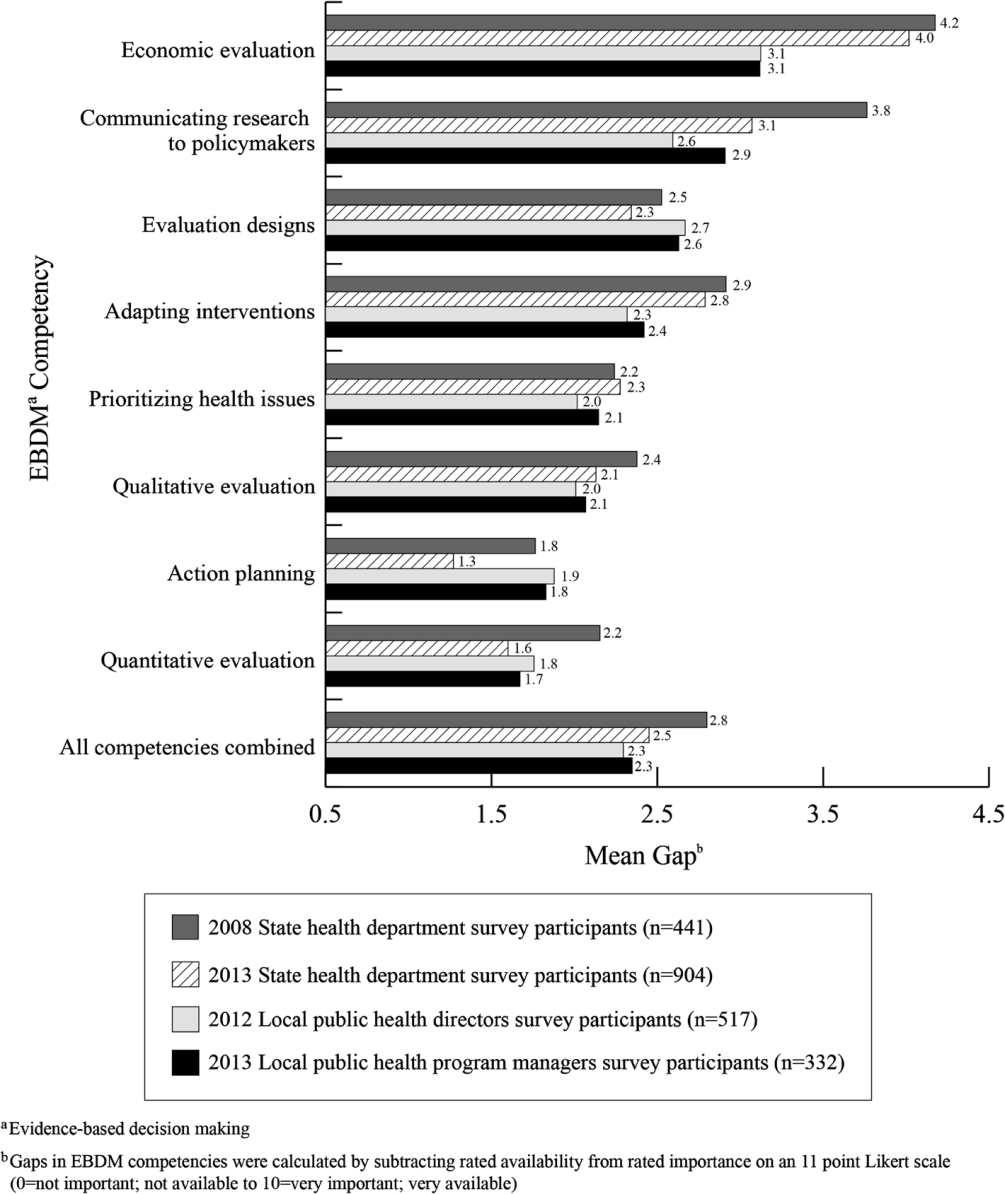
**Evidence-based decision making competency gaps in four national public health workforce surveys.** Staff in state and local public health departments were asked to rate importance and availability of competencies in evidence-based decision making (EBDM). Gaps in EBDM competencies were calculated by subtracting rated availability from importance and then aggregated for each survey.

Competencies with the largest training gaps were consistent across all four survey groups: economic evaluation, communicating research to policymakers, evaluation designs, and adapting interventions (Table 2). Table 3 shows mean importance, availability, and gap scores with corresponding 95% confidence intervals, p values from independent samples t tests, and Cohen’s d values for 2008 and 2013 state level workforce surveys. Importance and availability scores were significantly greater for state level staff in 2013 than in 2008 (p <0.001, d =1.00, and p <0.001, d =0.78 respectively). Participants from the 2013 state level survey reported significantly smaller mean gaps compared to those in 2008 (p <0.01, d =0.19). In addition, 2013 state level staff had significantly lower gaps in communicating research to policymakers (p <0.001, d =0.25), quantitative evaluation (p <0.001, d =0.23) and in action planning to achieve goals and objectives (p <0.001, d = 0.11) compared to state level staff in 2008. The overall mean importance score (8.8) and availability score (6.1) for the 2008 state level staff were the lowest among all four groups. The other three groups (2013 state level staff and both sets of local level participants) had similar mean importance and availability scores (mean importance range: 9.5 to 9.8 and mean availability range: 7.2 to 7.4) (data not shown). Figure 1 shows overall reported gaps for EBDM competencies were smaller for local public health staff than among state level staff.

**Table 2:** **Largest competency gaps in evidence-based decision making reported in four national public health workforce surveys**

**Gap rank** ^**a**^	**2008 State health department staff (n =441)**	**2013 State health department staff (n =904)**	**2012 Local public health directors (n =517)**	**2013 Local public health program managers (n =332)**
**1**	Economic evaluation	Economic evaluation	Economic evaluation	Economic evaluation
**2**	Communicating research to policymakers	Communicating research to policymakers	Evaluation designs	Communicating research to policymakers
**3**	Adapting interventions	Adapting interventions	Communicating research to policymakers	Evaluation designs
**4**	Evaluation designs	Evaluation designs	Adapting interventions	Adapting interventions

^a^Rank based on order of largest mean gap scores as calculated by subtracting rated availability from importance of EBDM competencies. A rank of “1” indicates the competency which had the largest mean gap.

**Table 3:** **Five-year comparison of state health department staff self-rated** ^**a**^ **EBDM competency importance, availability, and calculated gaps**

	**2008 State health department staff (n =441)** ^**b**^	**2013 State health department staff (n =904)** ^**b**^		
**Competency for evidence-based decision making**	**Mean (95% CI)**	**Mean (95% CI)**	***p*** ^**c**^	***d*** ^**d**^
Economic evaluation: *Understand how to use economic data in the decision making process.*				
Importance	8.8 (8.7-9.0)	9.7 (9.6-9.8)	<.001	0.52
Availability	4.7 (4.5-4.9)	5.6 (5.5-5.8)	<.001	0.36
Gap	4.2 (3.9-4.4)	4.0 (3.8-4.2)	.34	−0.05
Communicating research to policymakers: *Understand the importance of effectively communicating with policymakers about public health issues.*				
Importance	9.2 (9.1-9.3)	10.1 (10.0-10.2)	<.001	0.70
Availability	5.4 (5.2-5.6)	7.0 (6.8-7.2)	<.001	0.63
Gap	3.8 (3.5-4.0)	3.1 (2.9-3.3)	<.001	−0.25
Adapting Interventions: *Understand how to modify programs and policies for different communities and settings.*				
Importance	9.2 (9.1-9.3)	9.9 (9.8-10.0)	<.001	0.60
Availability	6.3 (6.0-6.5)	7.2 (7.0-7.3)	<.001	0.40
Gap	2.9 (2.7-3.1)	2.8 (2.6-3.0)	.37	−0.05
Evaluation designs: *Understand the different designs that are useful in program or policy evaluation.*				
Importance	8.2 (8.0-8.3)	9.7 (9.6-9.8)	<.001	0.96
Availability	5.6 (5.4-5.8)	7.4 (7.2-7.5)	<.001	0.72
Gap	2.5 (2.3-2.7)	2.3 (2.2-2.5)	.22	−0.07
Prioritization: *Understand how to prioritize program and policy options*				
Importance	8.9 (8.7-9.0)	9.9 (9.8-10.0)	<.001	0.73
Availability	6.7 (6.5-6.8)	7.6 (7.5-7.8)	<.001	0.45
Gap	2.2 (2.1-2.4)	2.3 (2.1-2.4)	.79	0.02
Qualitative evaluation: *Understand the value of qualitative evaluation approaches (e.g. focus groups, key informant interviews) including the steps involved in conducting qualitative evaluations.*				
Importance	8.5 (8.3-8.6)	9.5 (9.4-9.6)	<.001	0.68
Availability	6.1 (5.9-6.3)	7.4 (7.2-7.6)	<.001	0.55
Gap	2.4 (2.1-2.6)	2.1 (2.0-2.3)	.11	−0.10
Quantitative evaluation: *Understand the uses of quantitative evaluation approaches (e.g. surveillance and/or surveys).*				
Importance	8.6 (8.5-8.8)	9.9 (9.8-10.0)	<.001	0.83
Availability	6.5 (6.3-6.7)	8.3 (8.1-8.4)	<.001	0.76
Gap	2.2 (1.9-2.4)	1.6 (1.4-1.8)	<.001	−0.23
Action planning: *Understand the importance of developing an action plan for how to achieve goals and objectives.*				
Importance	9.2 (9.0-9.3)	10.2 (10.1-10.2)	<.001	0.82
Availability	7.4 (7.2-7.6)	8.9 (8.8-9.0)	<.001	0.73
Gap	1.8 (1.6-2.0)	1.3 (1.1-1.4)	<.001	−0.25
Overall average: *All EBDM competencies.*				
Importance	8.8 (8.7-8.9)	9.8 (9.8-9.9)	<.001	1.00
Availability	6.1 (5.9-6.2)	7.4 (7.3-7.5)	<.001	0.78
Gap	2.8 (2.6-3.0)	2.5 (2.3-2.6)	<.01	−0.19

*Abbreviations: EBDM* evidence-based decision making, *CI* confidence interval.

^a^Participants were asked to rate the “importance of each of the skills to you” then rate “how available each skill is to you when you need it” both on an 11 point Likert scale (0 = not important/available; 10 = very important/available). Gaps were calculated by subtracting the Likert score rating for availability from rated importance.

^b^Number represents the total number of participants in each survey; number of participants that responded to each competency varied slightly and calculations are for valid non-missing cases.

^c^P value for independent samples t test of mean differences in importance, availability, and gap scores between the 2008 and 2013 state health department participants.

^d^Cohen’s d calculated as d =2013 mean score minus the 2008 mean score and then divided by 2013 and 2008’s pooled standard deviation expressed as *d* = M_1_ - M_2_ / σ pooled. The direction (positive or negative) of d value is based on the input of 2013 mean scores first in the equation. Cohen suggests the following effect ranges: small 0.2; medium 0.5; and large 0.8.

### EBDM supports

**Figure 2:**
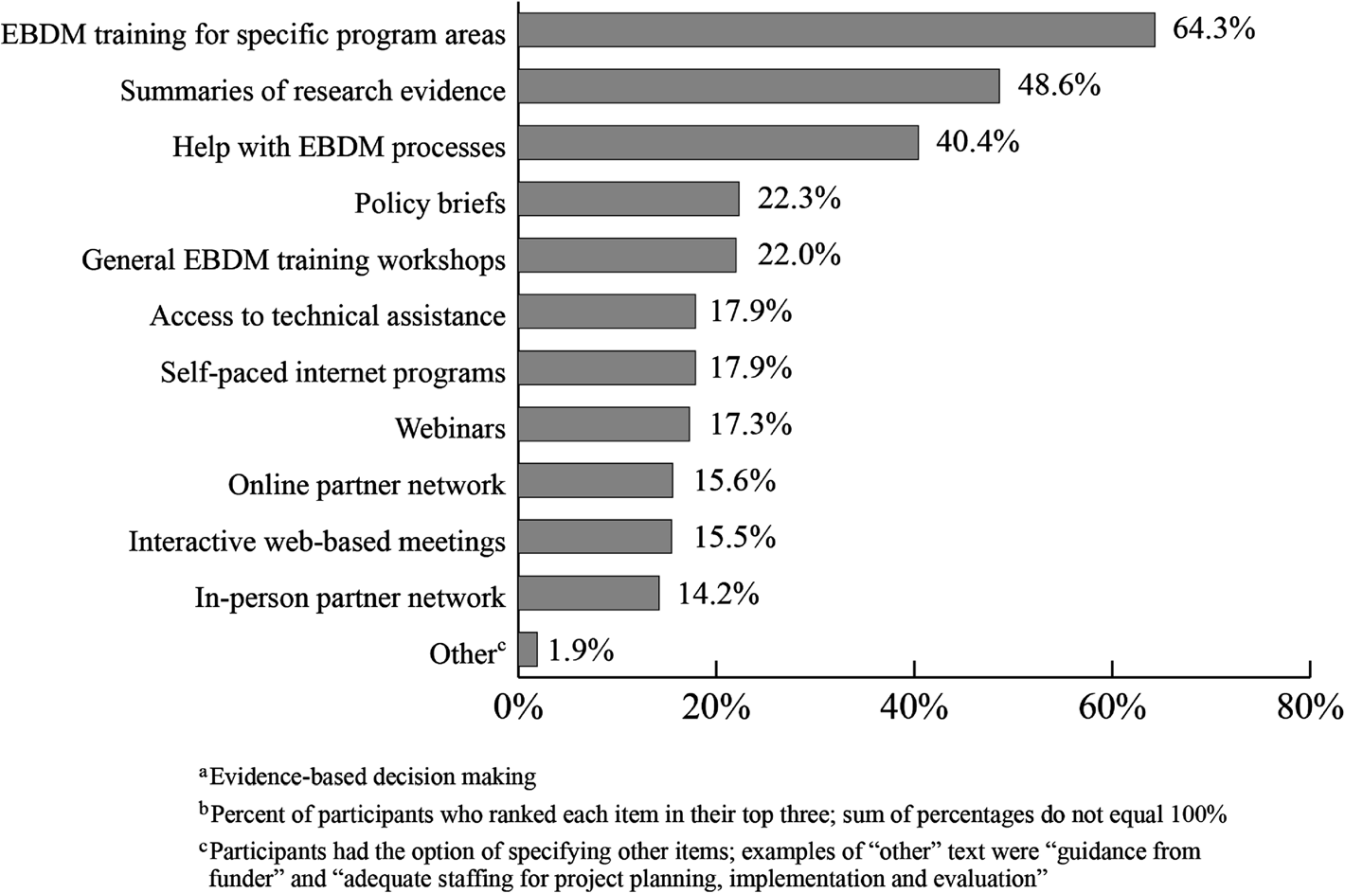
**Elements perceived by 2013 state health department staff as most useful in applying EBDM (n=904).** In 2013, state health department staff in all U.S. states was asked to rank their top three items which would be most useful for applying evidence-based decision making in their work. The percentage of participants who ranked each item among their top three was calculated.

Figure 2 shows items ranked by 2013 state health department staff as most useful for the application of EBDM. EBDM training for specific areas (64.3%), summaries of research evidence (48.6%), and help with EBDM processes (40.4%) were the top three most commonly ranked items. Two percent (1.9%) ranked an “other” item and filled in the supplied text box to represent an additional item not listed which would be useful for application of EBDM. Examples of “other” text are “guidance from funder” and “adequate staffing for project planning, implementation and evaluation.”

**Figure 3:**
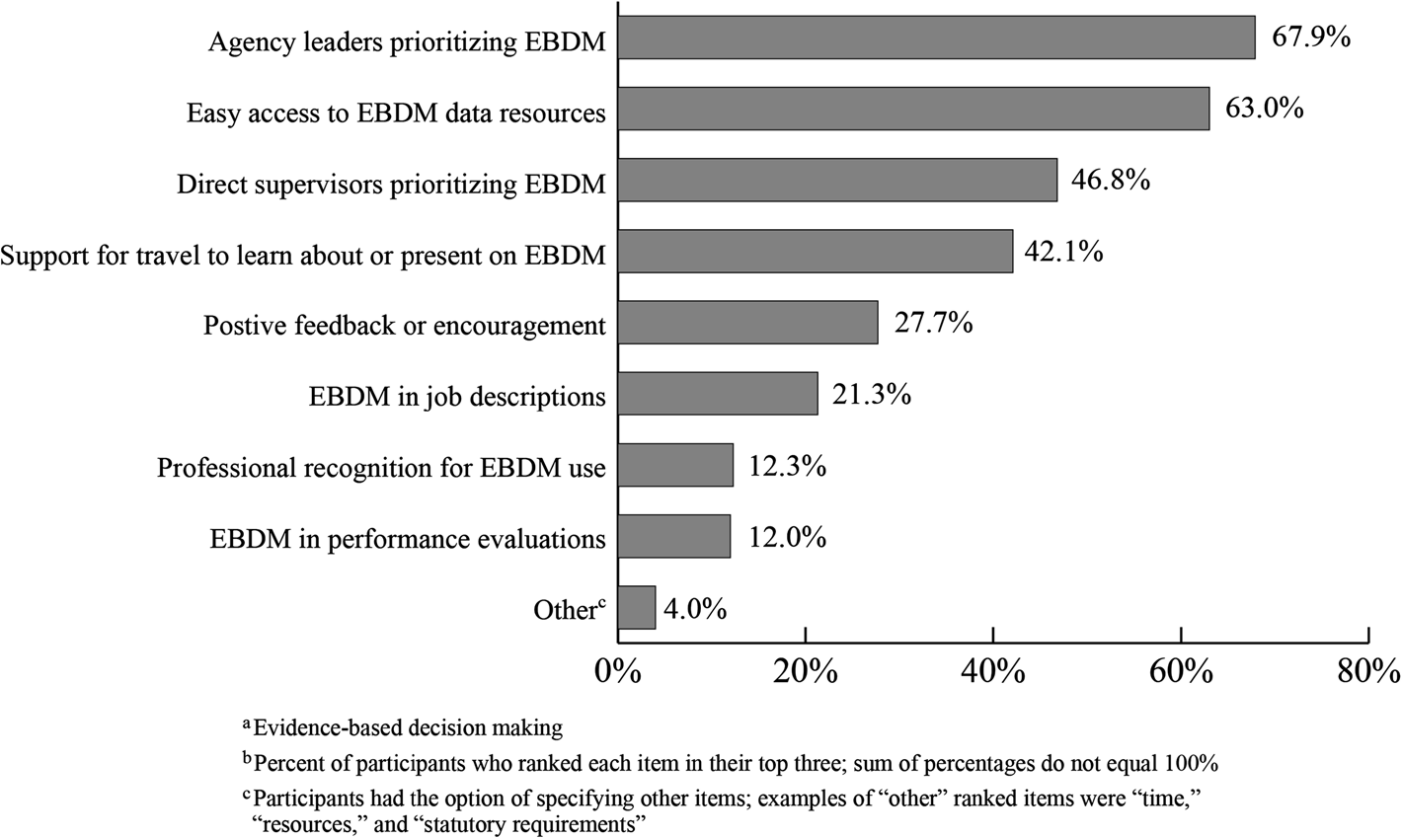
**Items ranked most important to encourage EBDM use by 2013 state health department staff (n =904).** In 2013, a national survey was conducted with state health department staff working in chronic disease. Participants were asked to rank their top three items which would be most important to encourage the use of evidence-based decision making. The percentage of participants who ranked each item among their top three was calculated.

Items to encourage use of EBDM that were most often ranked in the top three by the 2013 state level staff were agency leaders prioritizing EBDM (67.9%), easy access to EBDM data resources (63.0%), and direct supervisors prioritizing EBDM (46.8%) (Figure 3). Four percent of participants typed in and ranked “other” items that would encourage EBDM use. Examples of “other” ranked items were “time,” “resources,” and “statutory requirements”.

## Discussion

The public health workforce faces new and evolving challenges to both preventing and controlling disease in order to promote population health. This evolution requires an updated workforce and creates the need for specific training and skill sets not only for the topic areas in which people work, but also for the processes of applying the best, and integrating the newest, evidence. Overall, our findings suggest that competency to practice EBDM may be growing at the state public health department level and gaps in some skill areas (communicating research to policymakers, quantitative evaluation, and action planning) may be narrowing. However, the largest gaps remain in how to use economic evaluation data, adapt interventions, and design evaluations; and in communicating research to policymakers (despite improved scores in this area). Exact interpretation of the findings is difficult as possible reasons for narrowing gaps are likely many, complex, and compounded. Below, we offer some discussion of factors contributing to possible improvements followed by training needs indicated for the largest reported gaps.

### Growing supports for EBDM

Continued and growing trends to include requirements for and/or encouragement of EBDM processes in various public health funding streams may be a possible contributing factor for narrowing gaps and growing perceived availability of EBDM competencies. Many CDC funding announcements require grantees to use evidence-based processes through the implementation of evidence-based interventions, and in some cases, provide a menu of interventions to select from [40–43]. For example, the CDC Colorectal Cancer Screening Program requires grantees to implement evidence-based interventions to promote and increase colorectal cancer screening among underserved population groups, highlighting the need for application of evidence-based processes within health agencies [44]. Similarly, seeking PHAB accreditation may help to align activities of health departments with the requirements of funders. EBDM processes along with standardized certification measures, required for accreditation, may increase effective use of allocated funds, and possibly increase the likelihood of receiving further funding [45].

Along with funding trends, the growth of online tools and toolkits (e.g. Community Guide [12], Cancer P.L.A.N.E.T. [13], Research to Reality [14]) to aid and encourage EBDM processes has been expanded in the last five years, though the tools may not cover all competencies needed for EBDM. In addition, evidence-based public health courses which specifically target EBDM competencies are now offered by NACDD to two states each year as well as an annual national course which is open to applicants from chronic disease prevention from all U.S. states [46]. Similarly, other projects and schools of public health conduct comparable training with state and local public health agencies [17,19,47]. The Physical Activity and Public Health trainings held each September may also drive EBDM processes for applying evidence-based policies and programs specifically in the area of physical activity and obesity [48].

Related to increased training opportunities, another possible EBDM driving force may be growing numbers of public health programs and/or accreditation standards [49]. The Association of Schools and Programs of Public Health currently lists over 80 certified member schools and programs of public health which represent 39 U.S. states [50].This coupled with more evidence-based trainings may increase opportunity for collaborations between universities and public health agencies to exchange both science and practice evidence and offer increasing chances for exposure to EBDM processes. Academic partnerships to exchange practice and science-based evidence and ensure EBDM competencies may, however, be limited to agencies with geographic access to such universities. Consideration should be given to creating alternative models for long-distance partnering, such as video-conferencing and other communication technologies. The value of these collaborations has been overwhelmingly recognized by PHAB’s accreditation standards which require health departments to maintain academic partnerships as a means to “contribute to and apply the evidence base of public health” [9]. Even with growing numbers of undergraduate and graduate programs, the majority of the public health workforce lacks formal public health training [51,52]. Our findings show a range between 18% (2013 local level program managers) to 30% (2013 state level staff) of the workforce reported Master of Public Health degrees.

### Training needs for top competency gaps

Despite funders’ requirements and encouragement of evidence-based approaches, public health department employees continue to report competency gaps in several key areas (economic evaluation, communicating research to policymakers, adapting interventions, and evaluation designs). While not all, many of the identified gaps in EBDM competencies can be addressed through training programs. Thus, enhancing training capacity for these consistently reported gaps is necessary.

We found that competency to adapt interventions was among the largest gaps reported for all respondent groups and the gap did not narrow between 2008 and 2013 for the state level staff. While supplied intervention menus may be evidence-based (e.g. CDC’s Colorectal Cancer Screening Program), many health departments may be uncertain how to adapt them to ensure they have the expected impact given their population and context. This coincides with recent evaluation efforts of the National Comprehensive Cancer Control Program which found 78% of states reported the need for technical assistance in adapting evidence-based cancer interventions [53] and suggests more training is needed to build this EBDM competency.

Similarly, we found that economic evaluation was the largest reported gap in importance and availability among all four respondent groups. This parallels Wilcox and colleagues’ [54] recent study which found 66% of their sample of state, territorial, and local government public health professionals in chronic disease reported needing training in health economics. Understanding how to compare programs and policies based on economic costs and benefits is integral for the processes involved in making best decisions in public health. However, those formally trained in health economics have sparse knowledge of the unique demands of public health and are often lured outside the non-profit/governmental sphere or focus in medical care economics [55,56], making training specifically focused in this area challenging. Efforts to increase the number of health economists with public health programing knowledge may increase opportunities for agency-economist collaborations [57]. One such effort is CDC’s Prevention Effectiveness Fellowship program which trains post-doctoral candidates to assess policies and programs based on public health impact [58]. Similarly, increasing partnership opportunities between public health departments and persons with public health policy knowledge may help to improve competency in communicating with policymakers, which is imperative for effective feedback and framing of evidence-based policy [59].

In addition to increasing capacity for competency specific training, more attention to the tailoring of EBDM training and resources may be important as some competencies may simply be more difficult to master. Research in developing evidence-based competencies proposes three tiers (beginner, intermediate, and advanced) of difficulty level for EBDM competencies [8]. Of the top four consistent gaps found in this study, three (economic evaluation, communicating research to policymakers, and evaluation designs) are considered advanced and one intermediate (adapting interventions). While the national surveys in the current study largely featured those in a generalist or management position, large portions of the workforce practice at entry levels [6]. Improved training in EBDM and resource tailoring to diverse practice levels and backgrounds of the workforce, while adhering to adult learning frameworks, may help to further narrow EBDM competency gaps [6,30,60].

### Organizational opportunities to build capacity

While growing supports for EBDM competencies are suggested in the current study, it should be noted that EBDM as a process faces more than just the challenge of competency achievement or training. Organizational factors such as high staff turnover and the ability to sustain and make time for ongoing training for newly hired staff are commonly reported challenges to EBDM practice among public health agencies [61,62]. Evidence-based public health courses often last several days, requiring employees to reorganize busy schedules to accommodate training [17,61]. In addition, 2013 state level staff highly ranked “the prioritization of EBDM by superior staff” as a means of encouragement to use EBDM. This finding points to the importance of administrative practices that are likely to facilitate EBDM. For example, actively seeking and incorporating employee input into decision making processes and supervisory decisions to prioritize EBDM with provision of time to learn and engage in EBDM processes are administrative practices identified as means to create work environments conducive for EBDM [25,63].

### Limitations

Several limitations are worth noting. In the current study, we provide only self-report data from cross-sectional surveys. In addition, sampling methods and response rates for each survey varied, which may suggest response bias. For example, program managers identified by local health department directors for the 2013 local level survey may have been identified based on general responsiveness to emailed surveys, knowledge, or other unknown and varying factors. In addition, the two state level surveys included those working mainly in chronic disease prevention whereas the two local level surveys represented broader areas of public health practice (e.g. directors with overarching responsibilities, environmental health, and communicable disease prevention and control). This may limit our findings as we were not able to compare our local level data to state level data representing broader areas of practice outside of chronic disease prevention. Next steps for research should explore variations in capacity for EBDM across program areas (e.g., chronic disease compared with maternal and child health) and level of public health practice (e.g., state compared with local). In addition, this study did not explicitly examine health department characteristics, such as size and population jurisdiction, which have been shown to be associated with varying levels of department performance [35,64]. Further exploration of these characteristics is warranted given they are likely to influence capacity for EBDM and levels of competency. However, results for the largest reported EBDM competency gaps were consistent across all surveys. In the state surveys, it is difficult to ascertain the reasons for differences found in competency gaps over time. It is possible that the training and funding expectations have led to an increased awareness of the importance of EBDM but not necessarily the skills and competencies needed, suggesting the reduction in gap between importance and availability is really a function of bias due to socially desirable responses. Despite these limitations, this study offers an important national snapshot of EBDM competencies and their similarity across both the state and local level with corresponding implications for capacity development.

## Conclusions

Gaps in EBDM competencies may be narrowing as awareness and prioritization of skill development continue to grow through trends in funding, training, and resources for EBDM. However, across multiple sectors of the public health workforce, more capacity building is needed overall, with specific attention to the largest gaps in economic evaluation, communicating research to policymakers, adapting interventions to different settings and populations, and evaluation designs. More work with public health departments to both situate trainings to boost competency in these areas and continue to improve organizational practices are possible next steps to sustain EBDM efforts.
